# 2-(2-Nitro­anilino)-5,6,7,8-tetra­hydro-4*H*-cyclo­hepta­[*b*]thio­phene-3-carbonitrile

**DOI:** 10.1107/S1600536810017149

**Published:** 2010-05-15

**Authors:** Maria do Carmo A. de Lima, Francisco J. B. Mendonça Junior, Suely L. Galdino, Ivan R. Pitta, Carlos A. de Simone

**Affiliations:** a Laboratório de Síntese e Planejamento de Fármacos, Departamento de Antibióticos, Universidade Federal de Pernambuco, 50670-910 Recife, PE, Brazil; b Laboratório de Síntese e Vetorização de Moléculas Bioativas, Universidade Estadual da Paraíba, 58020-540 João Pessoa, PB, Brazil; c Departamento de Física e Informática, Instituto de Física de São Carlos, Universidade de São Paulo - USP, 13560-970 - São Carlos, SP, Brazil

## Abstract

The title compound, C_16_H_15_N_3_O_2_S, was synthesized by the reaction of 2-amino-5,6,7,8-tetra­hydro-4*H*-cyclo­hepta­[*b*]thio­phene-3-carbonitrile and *o*-fluoro­nitro­benzene. The thio­phene and nitro­phenyl rings and amino and carbonitrile groups are coplanar with a maximum deviation of 0.046 (2) Å and a dihedral angle of 0.92 (6)° between the rings. The cyclo­hepta ring adopts a chair conformation. Intra­molecular N—H⋯O and C—H⋯S inter­actions occur. In the crystal, the mol­ecules form layers that are linked by π–π stacking inter­actions between the thio­phene and benzene rings [centroid–centroid distances = 3.7089 (12) and 3.6170 (12) Å].

## Related literature

For background to 2-substituted thio­phenes, see: Campaigne (1984 ▶); Kleemann *et al.* (2006 ▶). For the biological activity of 2-amino thio­phene derivatives, see: Chakrabarti *et al.* (1982 ▶); Calligaro *et al.* (1997 ▶); Nikolakopoulos *et al.* (2006 ▶). For the synthesis of 2-amino thio­phenes, see: Gewald (1965 ▶); Gewald *et al.* (1966 ▶); Sridhar *et al.* (2007 ▶). For related structures, see: Stephenson *et al.* (1995 ▶); Yu (2002 ▶); Chen *et al.* (2005 ▶). For bond-length data, see: Allen *et al.* (1987 ▶). For puckering parameters, see: Cremer & Pople (1975 ▶).
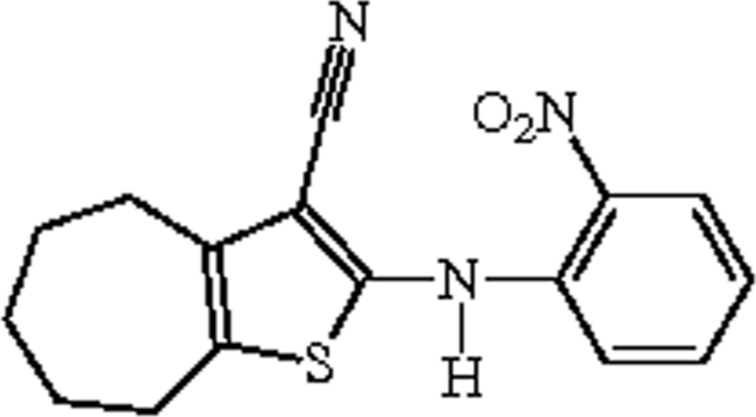


## Experimental

### 

#### Crystal data

C_16_H_15_N_3_O_2_S
*M* *_r_* = 313.37
Monoclinic, 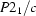

*a* = 7.0273 (3) Å
*b* = 14.4569 (6) Å
*c* = 14.8867 (7) Å
β = 97.571 (2)°
*V* = 1499.18 (11) Å^3^
*Z* = 4
Mo *K*α radiation
μ = 0.23 mm^−1^
*T* = 295 K
0.35 × 0.32 × 0.27 mm

#### Data collection

Nonius KappaCCD diffractometer
9360 measured reflections
3147 independent reflections
2452 reflections with *I* > 2σ(*I*)
*R*_int_ = 0.046

#### Refinement

*R*[*F* ^2^ > 2σ(*F* ^2^)] = 0.052
*wR*(*F* ^2^) = 0.162
*S* = 1.07
3147 reflections
200 parameters
H-atom parameters constrained
Δρ_max_ = 0.29 e Å^−3^
Δρ_min_ = −0.31 e Å^−3^

### 

Data collection: *COLLECT* (Nonius, 1997 ▶); cell refinement: *SCALEPACK* (Otwinowski & Minor, 1997 ▶); data reduction: *DENZO* (Otwinowski & Minor, 1997 ▶) and *SCALEPACK*; program(s) used to solve structure: *SHELXS97* (Sheldrick, 2008 ▶); program(s) used to refine structure: *SHELXL97* (Sheldrick, 2008 ▶); molecular graphics: *ORTEP-3 for Windows* (Farrugia, 1997 ▶); software used to prepare material for publication: *WinGX* (Farrugia, 1999 ▶).

## Supplementary Material

Crystal structure: contains datablocks I, global. DOI: 10.1107/S1600536810017149/dn2562sup1.cif

Structure factors: contains datablocks I. DOI: 10.1107/S1600536810017149/dn2562Isup2.hkl

Additional supplementary materials:  crystallographic information; 3D view; checkCIF report

## Figures and Tables

**Table 1:** Hydrogen-bond geometry (Å, °)

*D*—H⋯*A*	*D*—H	H⋯*A*	*D*⋯*A*	*D*—H⋯*A*
N1—H1⋯O1	0.86	1.89	2.593 (2)	138
C15—H15⋯S1	0.93	2.44	3.171 (2)	135

**Fig. 1.**
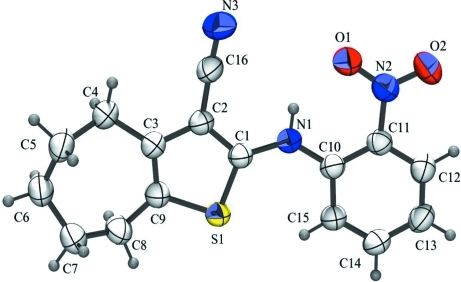
Molecular view of C16H15N3O2S, showing the atom labelling scheme. Ellipsoids are drawn at the 50% probability level. H atoms are represented as small spheres of arbitrary radii.

**Fig. 2.**
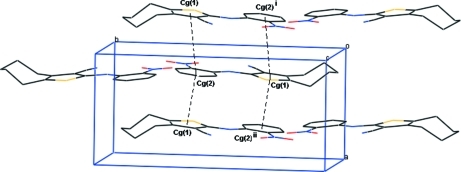
View showing π- π stacking interactions between the molecules. For the sake of clarity, H atoms have been omitted.

## supplementary crystallographic information

### Comment

The various uses of 2-substituted thiophenes have been well documented (Campaigne, 1984; Kleemann *et al.*, 2006). Amongst these appplications, are neuroleptics activities, as found for a series of thienobenzodiazepines (Chakrabarti *et al.*, 1982; Calligaro *et al.*, 1997; Nikolakopoulos *et al.*, 2006). In this work, we report the structure of the title compound prepared by the reaction of 2-amino-5,6,7, 8-tetrahydro-4*H*-ciclohepta[*b*]thiophene-3- carbonitrile and *o*-fluoro- nitrobenzene.

As indicated in the literature, compounds that presents *o*-nitrophenyl group linked to 2-amino-thiophene ring have great potential to produce crystalline structures, as found in 5-methyl-2-[(2-nitrophenyl)-amino]- thiophene-3-carbonitrile (ROY), that presents seven polymorphs coexisting at room temperature (Stephenson *et al.*, 1995; Yu, 2002; Chen *et al.*, 2005).

In the title compound,the least squares plane passing through all atoms of thiophene and nitrophenyl rings, and amino and carbonitrile groups, show planarity with maximum deviation of [0.046 (2) Å] for atom N3 (Fig.1). Bond lengths and angles are in good agreement with the expected values reported in the literature (Allen *et al.*, 1987). The cyclohepta ring adopts a chair conformation and the calculated puckering parameters are: q_2_ = 0.175 (9) Å, q_3_ = 0.619 (9) Å, Q_T_ = 0.643 (9) Å, θ = 15.8 (9)° , φ_2_ = 56.1 (8)° and φ_3_ = 78.4 (8)° (Cremer & Pople, 1975).

In the molecule there are, intramolecular N–H··· O, C—H··· O and C—H··· S interactions that are responsible for the roughly planar arrangement (Table 1). In the packing molecules form layers that extends along a direction parallel to the (100) plane, and are linked by π-π stacking interactions between thiophene [Cg1] and benzene [Cg2] rings(Table 2, Fig. 2).

### Experimental

To a stirred suspension mixture of dry THF (20 ml) and NaH (0,105 mol) at 0 °C under nitrogen was added dropwise a solution of 2-amino-5,6,7,8- tetrahydro-4*H*-ciclohepta[*b*]thiophene-3-carbonitrile (0,07 mol), *o*-fluoro-nitrobenzene (0,07 mol) in 80 ml of dry THF. The reaction mixture was stirred under room temperature for 22 h. The resulting mixture was adjusted to pH = 5 with hydroclorid acid 2 N and them extracted with CHCl_3_. The extracted was washed with aqueous Na_2_CO_3_, water, dried (CaCl_2_) and evaporated under reduced pression. The dark red solid obtained was purified by recrystallization from absolute ethanol, affording the title compound as red crystals (21.4 g, (98%), m.p. 100-102 °C)(Gewald, 1965; Gewald *et al.*, 1966; Sridhar *et al.*, 2007). Crystals suitable for single-crystal X-ray diffraction were grown by slow evaporation at room temperature of a solution of the pure title compound in ethanol/dichloromethane (1:1).

NMR ^1^H (200 MHz, CDCl_3_) δ : 1.60-1.70 (m, 4H), 1.80-1.90 (m, 2H), 2,70-2,80 (m, 4H), 6.90 (dt, 1H, *J* = 8.6, 1.6 Hz), 7.20 (dd, 1H, *J* = 8.6, 0.8 Hz), 7.5 (dt, 1H, *J* = 8.6, 8.2 Hz), 8.2 (dd, 1H, *J* = 8.4, 1.6 Hz), 9.6 (s, 1H).

NMR ^13^C (200 MHz, CDCl_3_) δ : 27.2-31.9 (5 CH_2_), 107.7, 113.9, 116.1, 119.5, 126.6, 133.9, 135.7, 136.1, 139.0, 141.4, 145.3.

### Refinement

All H atoms attached to C atoms and N atom were fixed geometrically and treated as riding with C—H = 0.93 Å (aromatic) or 0.97 Å (methylene) and N—H = 0.86 Å with U_iso_(H) = 1.2U_eq_(C or N).The maximum and minimum residual electron density peaks were located 1.10 and 0.78 Å, from the H4A and S1 atoms respectively.

### Crystal data

**Table:**

C_16_H_15_N_3_O_2_S	*F*(000) = 656
*M**_r_* = 313.37	*D*_x_ = 1.388 Mg m^−^^3^
Monoclinic, *P*2_1_/*c*	Mo *K*α radiation, λ = 0.71073 Å
Hall symbol: -P 2ybc	Cell parameters from 6392 reflections
*a* = 7.0273 (3) Å	θ = 2.9–26.7°
*b* = 14.4569 (6) Å	µ = 0.23 mm^−^^1^
*c* = 14.8867 (7) Å	*T* = 295 K
β = 97.571 (2)°	Prism, colorless
*V* = 1499.18 (11) Å^3^	0.35 × 0.32 × 0.27 mm
*Z* = 4	

### Data collection

**Table:**

Nonius KappaCCD diffractometer	2452 reflections with *I* > 2σ(*I*)
Radiation source: Enraf Nonius FR590	*R*_int_ = 0.046
horizonally mounted graphite crystal	θ_max_ = 26.6°, θ_min_ = 3.1°
Detector resolution: 9 pixels mm^-1^	*h* = −8→8
CCD rotation images,thick slices scans	*k* = −15→18
9360 measured reflections	*l* = −18→18
3147 independent reflections	

### Refinement

**Table:**

Refinement on *F*^2^	Primary atom site location: structure-invariant direct methods
Least-squares matrix: full	Secondary atom site location: difference Fourier map
*R*[*F*^2^ > 2σ(*F*^2^)] = 0.052	Hydrogen site location: inferred from neighbouring sites
*wR*(*F*^2^) = 0.162	H-atom parameters constrained
*S* = 1.07	*w* = 1/[σ^2^(*F*_o_^2^) + (0.1026*P*)^2^ + 0.1476*P*] where *P* = (*F*_o_^2^ + 2*F*_c_^2^)/3
3147 reflections	(Δ/σ)_max_ = 0.001
200 parameters	Δρ_max_ = 0.29 e Å^−^^3^
0 restraints	Δρ_min_ = −0.30 e Å^−^^3^

### Special details

**Table:**

Geometry. All esds (except the esd in the dihedral angle between two l.s. planes) are estimated using the full covariance matrix. The cell esds are taken into account individually in the estimation of esds in distances, angles and torsion angles; correlations between esds in cell parameters are only used when they are defined by crystal symmetry. An approximate (isotropic) treatment of cell esds is used for estimating esds involving l.s. planes.
Refinement. Refinement of *F*^2^ against ALL reflections. The weighted *R*-factor wR and goodness of fit *S* are based on *F*^2^, conventional *R*-factors *R* are based on *F*, with *F* set to zero for negative *F*^2^. The threshold expression of *F*^2^ > σ(*F*^2^) is used only for calculating *R*-factors(gt) etc. and is not relevant to the choice of reflections for refinement. *R*-factors based on *F*^2^ are statistically about twice as large as those based on *F*, and *R*- factors based on ALL data will be even larger.

### Fractional atomic coordinates and isotropic or equivalent isotropic displacement parameters (Å^2^)

**Table:**

	*x*	*y*	*z*	*U*_iso_*/*U*_eq_	
S1	0.29875 (7)	0.33887 (3)	0.05524 (3)	0.0529 (2)	
O1	0.1864 (3)	0.64817 (11)	−0.14472 (10)	0.0763 (5)	
O2	0.1729 (3)	0.78484 (12)	−0.09126 (12)	0.0887 (6)	
N1	0.2433 (2)	0.50661 (11)	−0.03762 (10)	0.0531 (4)	
H1	0.2277	0.5305	−0.0910	0.064*	
N2	0.1906 (2)	0.70191 (13)	−0.07994 (12)	0.0594 (4)	
N3	0.2128 (4)	0.44432 (17)	−0.26828 (13)	0.0871 (7)	
C1	0.2620 (2)	0.41167 (13)	−0.03697 (12)	0.0474 (4)	
C2	0.2544 (3)	0.36042 (14)	−0.11578 (12)	0.0501 (4)	
C3	0.2772 (3)	0.26261 (14)	−0.10156 (13)	0.0527 (5)	
C4	0.2731 (4)	0.19525 (16)	−0.17935 (14)	0.0673 (6)	
H4A	0.4041	0.1775	−0.1850	0.081*	
H4B	0.2221	0.2269	−0.2348	0.081*	
C5	0.1554 (3)	0.10721 (16)	−0.17147 (15)	0.0688 (6)	
H5A	0.0271	0.1251	−0.1609	0.083*	
H5B	0.1438	0.0750	−0.2291	0.083*	
C6	0.2345 (4)	0.04019 (15)	−0.09784 (16)	0.0707 (6)	
H6A	0.3688	0.0293	−0.1031	0.085*	
H6B	0.1677	−0.0183	−0.1086	0.085*	
C7	0.2190 (4)	0.07060 (16)	−0.00136 (16)	0.0710 (6)	
H7A	0.0872	0.0888	0.0021	0.085*	
H7B	0.2471	0.0179	0.0385	0.085*	
C8	0.3487 (4)	0.14916 (15)	0.03367 (16)	0.0669 (6)	
H8A	0.4798	0.1324	0.0270	0.080*	
H8B	0.3418	0.1564	0.0979	0.080*	
C9	0.3044 (3)	0.24118 (14)	−0.01205 (13)	0.0551 (5)	
C10	0.2447 (2)	0.57016 (13)	0.03089 (12)	0.0473 (4)	
C11	0.2192 (3)	0.66561 (13)	0.01206 (13)	0.0491 (4)	
C12	0.2185 (3)	0.73041 (15)	0.08074 (14)	0.0587 (5)	
H12	0.2012	0.7927	0.0663	0.070*	
C13	0.2430 (3)	0.70360 (16)	0.16918 (15)	0.0635 (5)	
H13	0.2427	0.7472	0.2151	0.076*	
C14	0.2683 (3)	0.61106 (16)	0.18993 (14)	0.0636 (5)	
H14	0.2845	0.5924	0.2503	0.076*	
C15	0.2700 (3)	0.54584 (15)	0.12264 (13)	0.0594 (5)	
H15	0.2884	0.4840	0.1386	0.071*	
C16	0.2285 (3)	0.40474 (15)	−0.20159 (13)	0.0594 (5)	

### Atomic displacement parameters (Å^2^)

**Table:**

	*U*^11^	*U*^22^	*U*^33^	*U*^12^	*U*^13^	*U*^23^
S1	0.0672 (3)	0.0504 (3)	0.0413 (3)	−0.00605 (19)	0.0074 (2)	0.00132 (18)
O1	0.1190 (14)	0.0599 (10)	0.0478 (8)	−0.0002 (8)	0.0034 (8)	0.0025 (7)
O2	0.1394 (16)	0.0503 (10)	0.0753 (11)	0.0042 (9)	0.0103 (10)	0.0123 (8)
N1	0.0707 (10)	0.0480 (9)	0.0407 (8)	−0.0010 (7)	0.0077 (7)	0.0000 (7)
N2	0.0684 (10)	0.0538 (10)	0.0558 (10)	−0.0019 (7)	0.0072 (8)	0.0042 (8)
N3	0.1274 (18)	0.0861 (15)	0.0481 (11)	0.0209 (13)	0.0123 (10)	0.0112 (10)
C1	0.0508 (9)	0.0485 (10)	0.0427 (9)	−0.0040 (7)	0.0061 (7)	0.0005 (7)
C2	0.0563 (10)	0.0523 (10)	0.0422 (9)	−0.0028 (8)	0.0078 (7)	0.0000 (8)
C3	0.0604 (10)	0.0505 (10)	0.0475 (10)	−0.0033 (8)	0.0084 (8)	−0.0032 (8)
C4	0.0929 (15)	0.0587 (12)	0.0513 (11)	−0.0070 (11)	0.0131 (10)	−0.0077 (10)
C5	0.0836 (14)	0.0569 (12)	0.0647 (13)	0.0012 (10)	0.0048 (11)	−0.0140 (10)
C6	0.0909 (16)	0.0494 (12)	0.0705 (14)	−0.0006 (11)	0.0057 (11)	−0.0047 (10)
C7	0.0915 (15)	0.0502 (12)	0.0715 (14)	−0.0034 (10)	0.0116 (11)	0.0040 (11)
C8	0.0859 (14)	0.0559 (13)	0.0566 (12)	−0.0038 (10)	0.0010 (10)	0.0049 (9)
C9	0.0650 (11)	0.0505 (11)	0.0497 (10)	−0.0063 (8)	0.0069 (8)	−0.0009 (8)
C10	0.0481 (9)	0.0511 (10)	0.0432 (9)	−0.0042 (7)	0.0081 (7)	−0.0011 (8)
C11	0.0493 (9)	0.0508 (11)	0.0474 (10)	−0.0045 (7)	0.0071 (7)	0.0001 (7)
C12	0.0653 (11)	0.0520 (11)	0.0597 (12)	0.0000 (9)	0.0120 (9)	−0.0070 (9)
C13	0.0725 (13)	0.0632 (13)	0.0569 (11)	−0.0048 (10)	0.0169 (9)	−0.0144 (10)
C14	0.0811 (13)	0.0665 (13)	0.0448 (11)	−0.0042 (10)	0.0147 (9)	−0.0043 (9)
C15	0.0786 (13)	0.0532 (11)	0.0478 (11)	−0.0023 (10)	0.0134 (9)	0.0008 (9)
C16	0.0750 (12)	0.0587 (12)	0.0442 (10)	0.0058 (9)	0.0066 (8)	−0.0043 (9)

### Geometric parameters (Å, °)

**Table:**

S1—C1	1.7220 (18)	C6—C7	1.520 (3)
S1—C9	1.735 (2)	C6—H6A	0.9700
O1—N2	1.236 (2)	C6—H6B	0.9700
O2—N2	1.215 (2)	C7—C8	1.506 (3)
N1—C10	1.372 (2)	C7—H7A	0.9700
N1—C1	1.379 (2)	C7—H7B	0.9700
N1—H1	0.8600	C8—C9	1.508 (3)
N2—C11	1.456 (2)	C8—H8A	0.9700
N3—C16	1.139 (3)	C8—H8B	0.9700
C1—C2	1.383 (2)	C10—C15	1.399 (3)
C2—C16	1.419 (3)	C10—C11	1.415 (3)
C2—C3	1.436 (3)	C11—C12	1.387 (3)
C3—C9	1.357 (3)	C12—C13	1.361 (3)
C3—C4	1.510 (3)	C12—H12	0.9300
C4—C5	1.531 (3)	C13—C14	1.379 (3)
C4—H4A	0.9700	C13—H13	0.9300
C4—H4B	0.9700	C14—C15	1.377 (3)
C5—C6	1.513 (3)	C14—H14	0.9300
C5—H5A	0.9700	C15—H15	0.9300
C5—H5B	0.9700		
			
C1—S1—C9	92.84 (9)	C8—C7—C6	115.5 (2)
C10—N1—C1	132.09 (16)	C8—C7—H7A	108.4
C10—N1—H1	114.0	C6—C7—H7A	108.4
C1—N1—H1	114.0	C8—C7—H7B	108.4
O2—N2—O1	121.38 (19)	C6—C7—H7B	108.4
O2—N2—C11	119.03 (18)	H7A—C7—H7B	107.5
O1—N2—C11	119.59 (17)	C7—C8—C9	115.43 (19)
N1—C1—C2	122.32 (17)	C7—C8—H8A	108.4
N1—C1—S1	128.18 (14)	C9—C8—H8A	108.4
C2—C1—S1	109.50 (14)	C7—C8—H8B	108.4
C1—C2—C16	120.52 (18)	C9—C8—H8B	108.4
C1—C2—C3	114.31 (17)	H8A—C8—H8B	107.5
C16—C2—C3	125.17 (18)	C3—C9—C8	129.69 (19)
C9—C3—C2	111.61 (17)	C3—C9—S1	111.73 (15)
C9—C3—C4	126.28 (19)	C8—C9—S1	118.46 (15)
C2—C3—C4	122.11 (18)	N1—C10—C15	123.03 (18)
C3—C4—C5	115.66 (17)	N1—C10—C11	121.17 (16)
C3—C4—H4A	108.4	C15—C10—C11	115.79 (17)
C5—C4—H4A	108.4	C12—C11—C10	121.68 (18)
C3—C4—H4B	108.4	C12—C11—N2	115.89 (18)
C5—C4—H4B	108.4	C10—C11—N2	122.43 (16)
H4A—C4—H4B	107.4	C13—C12—C11	120.6 (2)
C6—C5—C4	115.93 (19)	C13—C12—H12	119.7
C6—C5—H5A	108.3	C11—C12—H12	119.7
C4—C5—H5A	108.3	C12—C13—C14	119.26 (19)
C6—C5—H5B	108.3	C12—C13—H13	120.4
C4—C5—H5B	108.3	C14—C13—H13	120.4
H5A—C5—H5B	107.4	C15—C14—C13	120.97 (19)
C5—C6—C7	115.73 (19)	C15—C14—H14	119.5
C5—C6—H6A	108.3	C13—C14—H14	119.5
C7—C6—H6A	108.3	C14—C15—C10	121.7 (2)
C5—C6—H6B	108.3	C14—C15—H15	119.1
C7—C6—H6B	108.3	C10—C15—H15	119.1
H6A—C6—H6B	107.4	N3—C16—C2	176.3 (2)

### Hydrogen-bond geometry (Å, °)

**Table:**

*D*—H···*A*	*D*—H	H···*A*	*D*···*A*	*D*—H···*A*
N1—H1···O1	0.86	1.89	2.593 (2)	138
C12—H12···O2	0.93	2.33	2.657 (3)	100
C15—H15···S1	0.93	2.44	3.171 (2)	135

### Table 2 π–π stacking interactions (Å, °) between the thiophene (Cg1) and benzene (Cg2) rings.

**Table:**

	Cg1···Cg2	Cg1 to plane 2	Cg2 to plane 1	Offset
Cg1···Cg2^i^	3.7089 (12)	-3.5039 (9)	-3.5021 (9)	19.4
Cg1···Cg2^ii^	3.6170 (12)	3.4761 (9)	3.4878 (9)	16.0

Symmetry codes: (i) -x, 1-y, -z; (ii) 1-x, 1-y, -z.
